# Phosphate-solubilizing microorganisms for soil health and ecosystem sustainability: a forty-year scientometric analysis (1984–2024)

**DOI:** 10.3389/fmicb.2025.1546852

**Published:** 2025-02-19

**Authors:** Yiming Lei, Yuhan Kuai, Mingyu Guo, Huan Zhang, Yuan Yuan, Hualong Hong

**Affiliations:** ^1^Key Laboratory of the Ministry of Education for Coastal and Wetland Ecosystems, Xiamen University, Xiamen, China; ^2^College of Science, Yunnan Agricultural University, Kunming, China; ^3^Key Laboratory of Western China’s Environmental Systems (Ministry of Education), College of Earth and Environmental Sciences, Center for Glacier and Desert Research, Lanzhou University, Lanzhou, China; ^4^College of Resources and Environment, Huazhong Agricultural University, Wuhan, China

**Keywords:** phosphorus availability, ecological restoration, crop growth, global research trends, sustainable agriculture, microbial soil nutrients

## Abstract

Phosphate-solubilizing microorganisms (PSM) play a crucial role in promoting crop growth by enhancing phosphorus supply and reducing phosphorus loss in soil. However, a comprehensive bibliometric overview of the research landscape on PSM in agricultural applications has been lacking. This study conducts a bibliometric analysis to explore global research trends, key contributors, and collaborative networks in the application of PSM in ecological restoration, providing valuable insights for future research. A total of 1,662 documents from the Web of Science Core Collection, spanning from 1984 to 2024, were extracted and analyzed using Bibliometrix and CiteSpace software. The findings reveal a period of rapid growth in this field since 2018. Initially, research focused on microbial soil nutrients, such as phosphate rock and *Azospirillum brasilense*. Current research hotspots have shifted towards topics like drought and salt stress, as well as productivity, reflecting an increasing emphasis on mitigating the impacts of global warming and environmental changes. China and India lead in research output, contributing 36.67% of the total articles. The Indian Council of Agricultural Research published the highest number of articles. Future research on PSM should emphasize their role in enhancing nutrient uptake, improving soil health, and mitigating environmental stresses, supporting sustainable agriculture and ecological restoration. This bibliometric analysis of 1,162 articles by 7,454 authors from 101 countries highlights critical advances at the intersection of soil microbiology, sustainable land management, and climate change adaptation. These findings provide a foundation for addressing global challenges like soil degradation, nutrient cycling, and food security, aligning with the Sustainable Development Goals.

## 1 Introduction

Phosphorus is a critical macronutrient essential for plant growth and development, playing a pivotal role in various processes including energy transfer, photosynthesis, and nutrient cycling (Kamerlin et al., 2013; Majeed et al., 2023). Despite its significance, phosphorus often acts as a limiting factor in numerous terrestrial ecosystems, primarily due to its existence in forms that are not readily available to plants (Syers et al., 2008; Rawat et al., 2021). Global estimates indicate that approximately 40% of the world’s agricultural soils suffer from phosphorus deficiency, which negatively impacts both food production and ecosystem health (Vance et al., 2003). This limitation severely hampers soil productivity and the capacity for ecosystem recovery, posing a significant challenge to sustainable land management and ecological restoration efforts (Xiao et al., 2021; Wang et al., 2023). In regions where phosphorus depletion is most acute, soil fertility often declines by over 50%, thereby greatly restricting the potential for ecosystem recovery (Huang et al., 2012; Qin et al., 2023). A promising approach to address phosphorus deficiency in soils involves the utilization of phosphate-solubilizing microorganisms (PSM) (Richardson and Simpson, 2011). As a crucial component of soil microbiota, PSM can convert insoluble soil phosphorus into plant-accessible forms through their metabolites (organic acids, phosphatases) or via synergistic interactions within microbial communities (Richardson et al., 2009; Alori et al., 2017; Rawat et al., 2021). This not only improves phosphorus uptake by plants, but also accelerates nutrient cycling, enhances soil structure, and supports broader ecosystem functions by accelerating nutrient cycling and improving the bioavailability of critical minerals, making it a key strategy for sustainable ecosystem management (Rajwar et al., 2018).

The role of PSM in ecological restoration has gained prominence due to their capacity to enhance nutrient cycling, stimulate plant growth, and aid biodiversity recovery in degraded ecosystems (Liang et al., 2020; Chen et al., 2023). By improving phosphorus availability in the soil, By increasing phosphorus bioavailability, PSM contribute to critical ecosystem services, such as improving soil stability, supporting vegetation regrowth, and enhancing carbon sequestration (Sun et al., 2023; Chen et al., 2024). These attributes align closely with global efforts to address land degradation and promote sustainable land management (Hussain et al., 2019; Silva et al., 2023; Gurav et al., 2024). Furthermore, In the context of global warming, PSM have shown significant potential to help ecosystems adapt to changing climatic conditions by enhancing nutrient cycling under stress conditions, such as drought and heat (Aqeel et al., 2023). This ability to maintain or restore ecosystem functions under climate change is crucial for increasing ecosystem resilience, ensuring food security, and mitigating climate-related impacts on ecosystems (Rawat et al., 2021).

While the ecological and agricultural importance of PSM has been extensively studied (Lambers, 2022; Iftikhar et al., 2024), a systematic and comprehensive global analysis of this research domain remains largely unexplored (Alori et al., 2017; Ampese et al., 2022). Existing studies often neglect the evolving research priorities and their implications for land management and ecosystem restoration, limiting a holistic understanding of PSM’s role in sustainability. Bibliometrics involves the quantitative analysis of academic publications and their citation data to comprehend patterns and developments in scientific knowledge dissemination (Garfield, 1972; Aria and Cuccurullo, 2017; Ampese et al., 2022; Montazeri et al., 2023). Its primary objectives include measuring research output impact, evaluating academic contributions of institutions or individuals, identifying research trends, and monitoring the evolution of scholarly domains (Glänzel, 2003; Wang et al., 2024). A fundamental tool in bibliometrics is citation analysis, which assesses the significance of research based on citation frequency (Van Raan, 1997). Collaboration network analysis elucidates relationships among scholars, facilitating understanding of scientific cooperation and knowledge dissemination patterns (Aria and Cuccurullo, 2017). Beyond identifying research hotspots, bibliometrics tracks disciplinary development, providing empirical support for research evaluation, academic management, and policy formulation, ultimately contributing to the optimal allocation of research resources (Egghe and Rousseau, 1990; Hirsch, 2010).

This study aims to conduct a bibliometric analysis on the application of PSM in ecological restoration, utilizing data from the Web of Science Core Collection, covering literature from 1984 to 2024. Through an examination of publication trends, author collaborations, keyword co-occurrence, and geographic distribution, this research endeavors to map the development of PSM research within the context of ecological restoration. The findings aim to provide a comprehensive landscape of PSM research, identifying significant contributors, uncovering emerging themes, and pinpointing underexplored areas that can guide future ecological restoration strategies. This analysis intends to provide researchers and practitioners with insights for more effective utilization of PSM in ecological restoration efforts.

## 2 Materials and methods

### 2.1 Data sources and preprocessing

**Figure 1:**
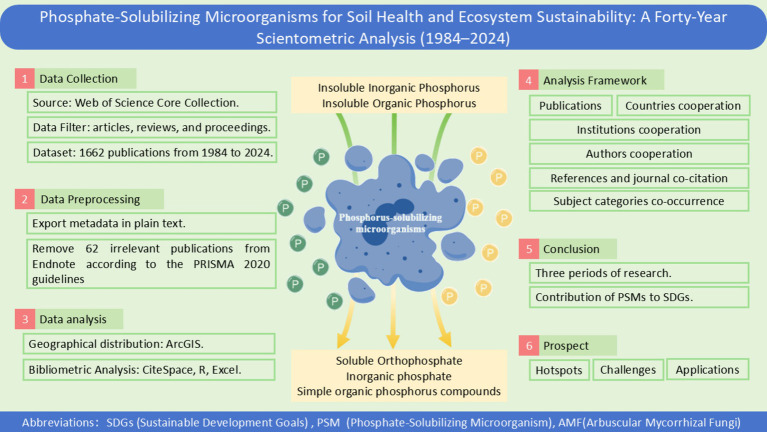
Bibliometric analysis flow of this study. Abbreviation in this study: SDGs (Sustainable Development Goals), PSM (Propensity Score Matching) and AMF (*Arbuscular Mycorrhizal* Fungi).

As shown in Figure 1, This study was conducted according to the PRISMA 2020 guideline (Page et al., 2021). The search was conducted in “Web of Science Core Collection,” with “All” selected in “Editions.” The search query focused primarily on “Phosphate-solubilizing microorganisms,” and the data retrieval strategies were designed as follows: TS = (“phosphor-releas*” OR “phosphor-solubili*” OR “phosphate-releas*” OR “phosphate-solubili*” OR “phosphor-mobiliz*” OR “phosphate-mobiliz*” OR “phosphor releas*” OR “phosphor solubili*” OR “phosphate releas*” OR “phosphate solubili*” OR “phosphor mobiliz*” OR “phosphate mobiliz*”) AND TS = (microorganism* OR microb* OR bacteria* OR Fungi OR Fungus) AND TS = (ecolog* OR ecosystem* OR environment* OR nutrient* OR fertilit*) AND TS = (rehabilit* OR restor* OR improve* OR remedia* OR bioremedia* OR sustainab* OR manage*). The data used in this study can be accessed through Mendeley Data (Mendeley Data, V2, doi:10.17632/xrp4hp58gs.2).

This initial query yielded 1,378 English publications on December 31, 2023, covering the period from January 1, 1994, to December 31, 2023. An additional search was conducted on December 9, 2024, extending the dataset to 1,662 publications. After screening the records (Figure 1), 47 records were excluded due to incomplete bibliographic information, such as missing author names, publication dates, or other essential details necessary for proper analysis. Following full-text review, 15 records were excluded because they were not relevant to the content of the study.

### 2.2 Data analysis

To analyze the retrieved data comprehensively, this study utilized CiteSpace (6.3.R1 Advanced), Bibliometrix R-package (Aria and Cuccurullo, 2017), and Microsoft Excel 2021. These tools were selected based on their established effectiveness in conducting co-occurrence analysis, collaboration network mapping, and visualizing research trends. CiteSpace was used for co-citation and keyword co-occurrence analyses, identifying research hotspots and trends over time (Chen and Leydesdorff, 2014; Chen, 2017). These analyses revealed correlations between literature, emerging frontiers, and thematic developments (Chen, 2006; Montazeri et al., 2023; Du and Li, 2024). CiteSpace’s algorithms were also applied to assess keyword characteristics, where node size indicated frequency, and link thickness reflected co-occurrence strength (Li et al., 2023). Key nodes (centrality >0.1) were identified as critical to connecting research themes, and the burstiness indicator highlighted emerging research trends and hotspots (Guo and Yao, 2024). Pajek complemented this by processing large-scale network data, enabling visualization of collaboration and citation networks (Batagelj and Mrvar, 2004). ArcGIS facilitated spatial analyses to map research distributions and academic cooperation patterns (Borge-Holthoefer and Moreno, 2012). Bibliometrix provided a robust platform for bibliometric analysis, integrating data transformation, analysis, and visualization (Aria and Cuccurullo, 2017).

However, bibliometric methods may introduce certain biases. Data source selection can lead to sample bias, as reliance on databases like Web of Science or Scopus may overlook non-English and regional studies (Alois, 2024). Additionally, keyword extraction and classification involve subjectivity, where handling synonyms and polysemes can affect results (Liu et al., 2021). Overemphasis on highly cited papers may also overshadow emerging or niche research areas and these biases can impact the accurate identification of research hotspots and trends (Larivière, 2010). In this study, all the literature data have been optimized to avoid the above problems.

## 3 Results

### 3.1 Publication analysis

**Figure 2:**
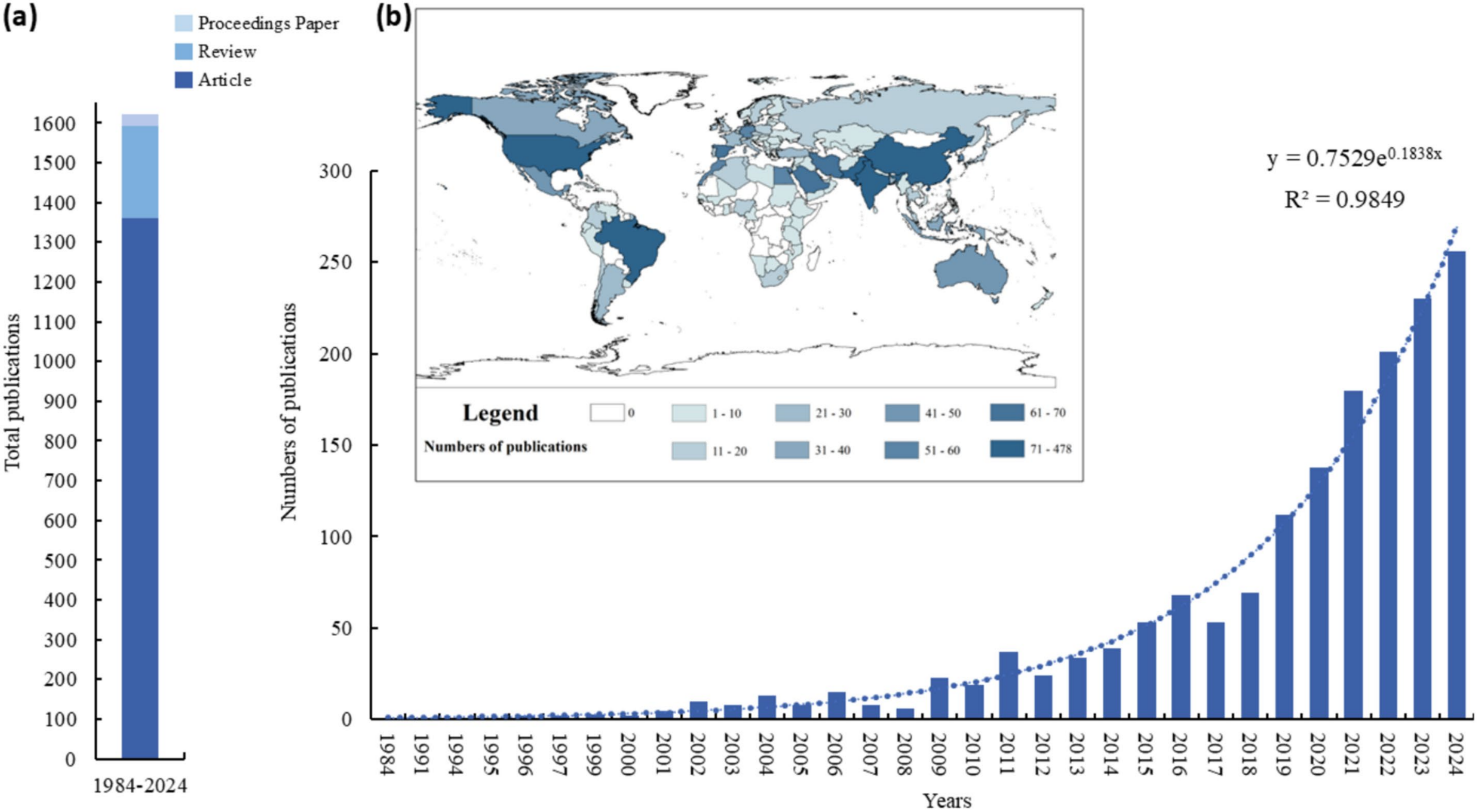
Publication output in the field of PSM from 1984 to 2023. **(A)** Stack chart of total publications. **(B)** Annual and cumulative number of publications and geographic visualizations.

The number of publications indicates the total count of papers published within a specific research field over a designated period, and fluctuations in this number can signify development trends within that field (Pan et al., 2024). The corpus of publications, as shown in Figure 2 (a), primarily comprised articles (1367), followed by reviews (233), and proceedings papers (17). The publication trend can be divided into three stages (1984–2008, 2009–2017, 2018 to present) as illustrated in Figure 2 (b). In the first stage, the quantity of publications was relatively low and grew slowly. The annual number of publications remained in single digits to approximately 10. The first two articles were published in 1984 and 1991, representing the initial stage of the PSM studies. During this period, the research field was gradually building and the growth was steady but modest, reflecting the early development phase of the field. From 2008, the number of publications began to gradually increase. By 2017, the number of publications exceeded 50. The years 2006 and 2009 exhibited two small “peaks,” with growth rates of 90.91 and 116.67%, respectively. These peaks indicate periods of increased research activity and interest in the field. The growth during this period was more pronounced compared to the initial stage. From 2018 onwards, the number of publications increased significantly. The peak number of publications was reached in 2021 and 2022, at nearly 160. In 2019, the number of publications increased by 58.06% compared to 2018, and in 2021, it increased by 38.46% compared to 2020. This sharp increase in publications reflects the rapid growth of research in the field, driven by advancements in technology, increased funding, and a growing recognition of the importance of PSM studies in addressing contemporary challenges.

Based on the cumulative number of publications per year, an equation for fitting the curve can be derived: y = 0.7529e^0.1838x^, *R*^2^ = 0.9849, demonstrating excellent fitting properties and conforming to the Price curve. This trend is particularly evident in recent years, with research potentially related to ecological restoration and PSM garnering increasing attention.

### 3.2 Countries cooperation analysis

From a bibliometric perspective, 101 countries have conducted research on PSM, and 21 countries have published more than 20 articles each. Supplementary Table S1 lists the top 20 countries by number of published papers. Three countries have surpassed the threshold of 100 publications, collectively accounting for 41.47% of the total publications. India leads with 478 publications, closely followed by China with 349. Notably, India initiated research in this field earlier, and the first two articles published in 1984 and 1991 were both from India. Pakistan ranks third with 130 publications.

**Figure 3:**
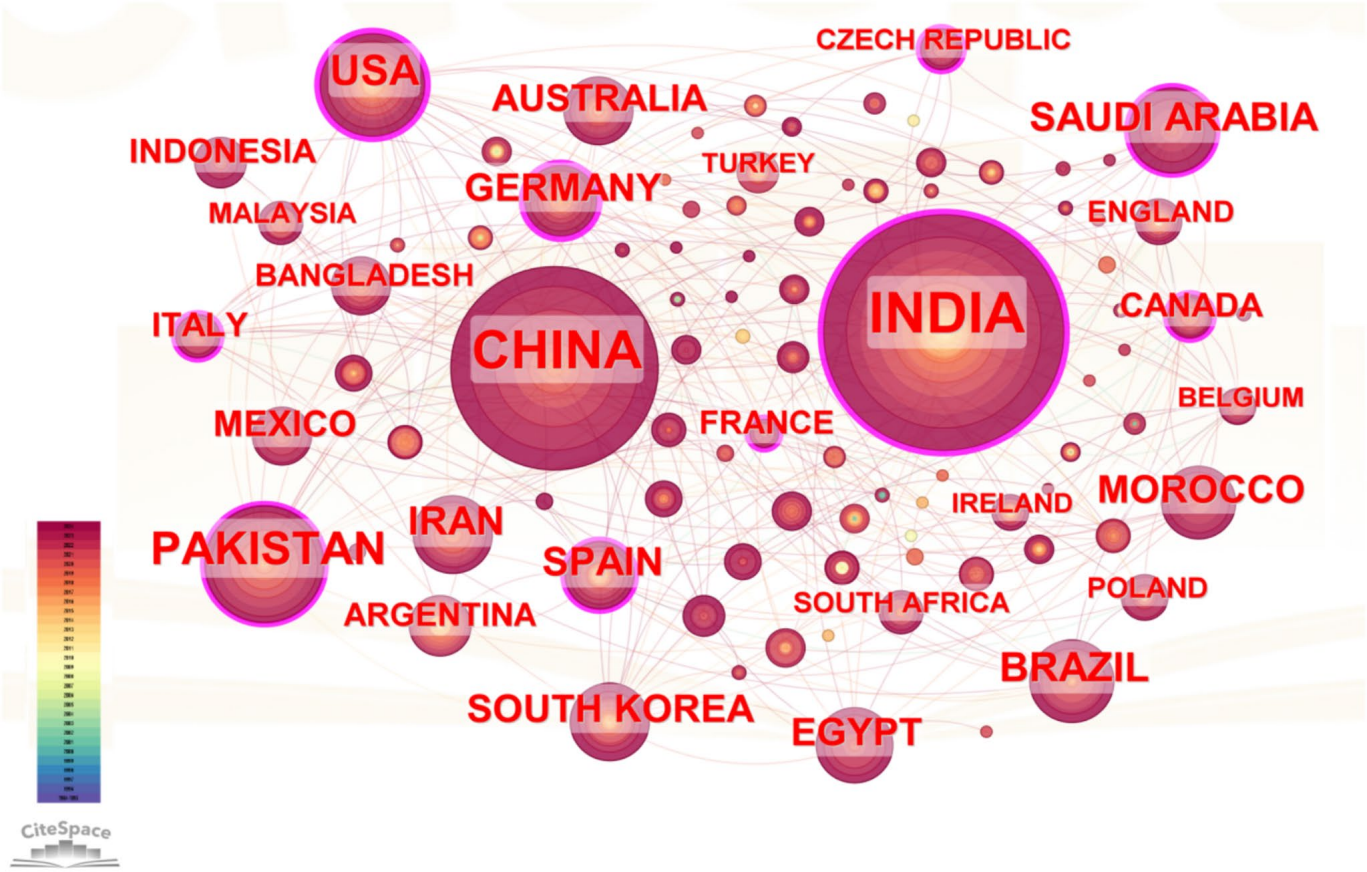
Map of country cooperation network analysis of PSM publications. Nodes represent countries involved in the collaboration network, with size indicating their level of participation. Colors reflect collaboration frequency, with darker nodes showing more active partnerships. Lines represent collaborations between countries, with thicker lines indicating stronger or more frequent ties.

In the collaboration network shown in Figure 3, India, China, and Pakistan are prominently positioned with larger nodes, signifying their high publication output. India’s dominant role is further emphasized by its extensive connections with numerous countries, reflecting strong international collaborations. China also exhibits robust collaborative links, particularly with the USA, Germany, and Australia, highlighting research partnerships. France’s high centrality suggests it acts as a key bridge in the network, facilitating collaboration between other major research hubs. Countries like Germany, Iran, and South Korea display moderate centrality, implying active participation but with less influence compared to France and the USA. Clusters of closely connected countries are evident, such as the collaborations between European nations (France, Germany, Spain, and Italy) and between Asian countries (India, China, Pakistan, and Bangladesh). These clusters suggest regional research networks that complement broader international collaborations. Overall, the visualization in Figure 3 demonstrates a well-integrated global research network on PSM, characterized by strong bilateral and multilateral partnerships. This interconnected framework facilitates knowledge sharing and accelerates advancements in sustainable agricultural technologies and ecosystem restoration initiatives.

Co-occurrence network analysis of research topics in the field of PSM and national collaborations reveal seven distinct thematic clusters (Supplementary Figure S1), showing connections between countries and research themes. Cluster #0 (soil fertility), dominated by India and Australia, focuses on improving soil nutrient availability. Cluster #1 (growth-promoting rhizobacteria), linked to Pakistan and Brazil, emphasizes enhancing plant growth. Cluster #2 (oil palm tree), represented by Indonesia, explores PSM applications in tropical agriculture. Cluster #3 (calcareous soil), involving Pakistan and Spain, addresses challenges in alkaline soils. Cluster #4 (phosphorus deficiency), associated with China and the USA, investigates solutions for phosphorus scarcity. Cluster #5 (unlocking agro-ecosystem sustainability), connected to Italy and France, focuses on sustainable farming, while Cluster #7 (environmental remediation), linked to Germany, highlights PSM’ role in restoring degraded environments. This network reflects the global collaboration in advancing PSM research for sustainable agriculture.

### 3.3 Institution cooperation analysis

The distribution of collaboration and beneficial contribution among diverse institutions in the research field is analyzed using CiteSpace. In the selection criteria, the scale factor k = 5. The analysis revealed that 2097 institutions published research articles in the field of PSM, and the top 20 institutions with the highest number of publications are presented in Supplementary Figure S2. The results indicate that the Indian Council of Agricultural Research (India) ranks first with 111 papers, followed by the Egyptian Knowledge Bank (Egypt) and Chinese Academy of Sciences (China), each with more than 40 publications. Among the top 20 institutions in terms of publication count, India leads with 259, followed by China with 74 and Morocco with 55. The three institutions with the highest centrality are Bahauddin Zakariya University (Pakistan), Huazhong Agricultural University (China), and Al Azhar University (Egypt), suggesting their relatively high comprehensive influence in this field.

**Figure 4:**
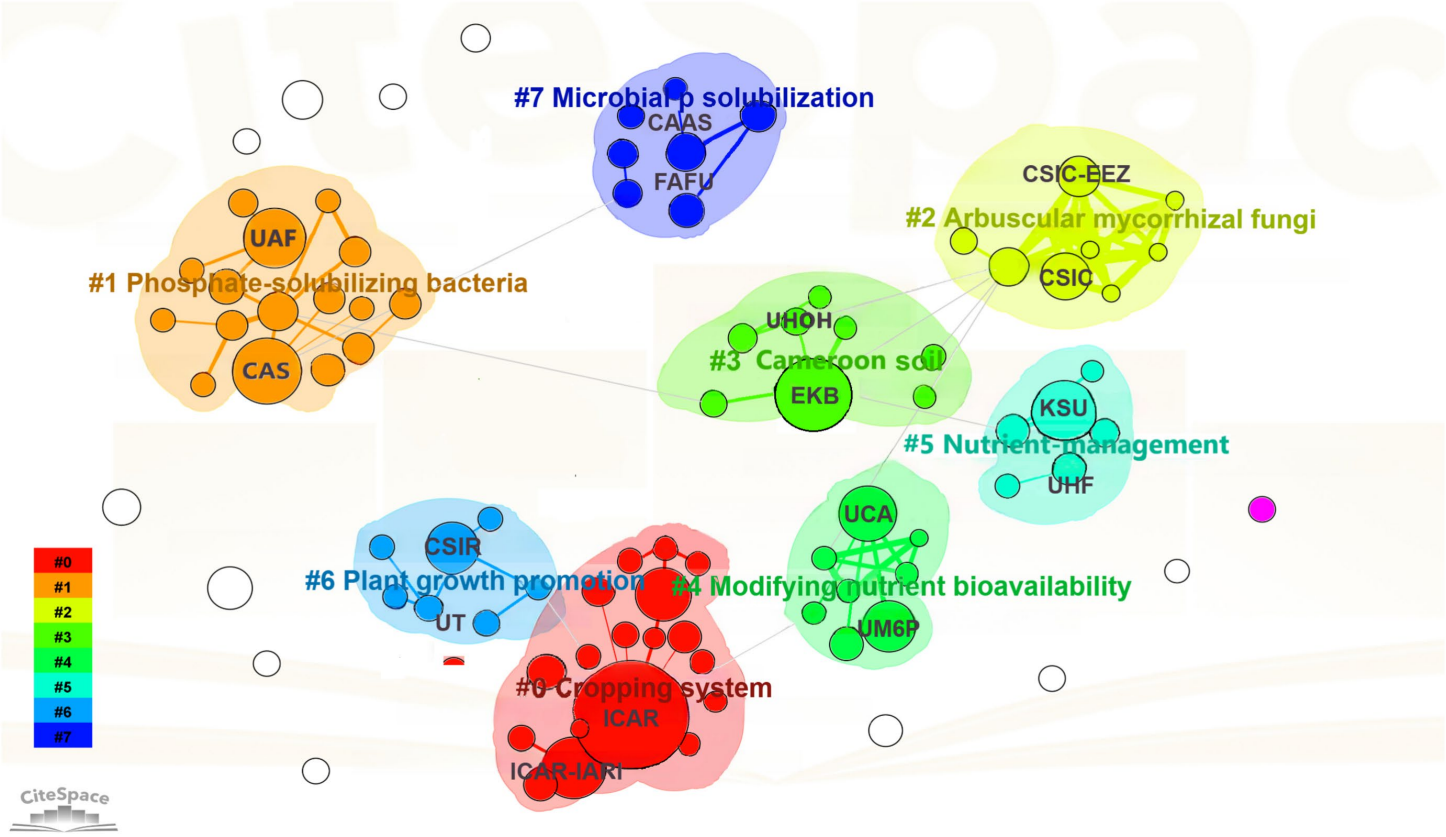
Map of collaborative network of global institutions. Colors represent different research clusters. Nodes indicate research institutions, with node size reflecting their influence, and edges represent collaboration links between institutions.

The collaborative network of research institutions in the field of PSM highlight the relationship and contributions of key institutions (Figure 4). The full names, abbreviations and countries of these institutions are shown in Table 1. Cluster #0 (Cropping System) is dominated by the Indian Council of Agricultural Research (India) and the Indian Agricultural Research Institute (India), which play central roles in advancing sustainable farming practices. Cluster #1 (Phosphate-Solubilizing Bacteria) features close collaboration between the Chinese Academy of Sciences (China) and the University of Agriculture Faisalabad (Pakistan), emphasizing research on microbial solutions for nutrient availability. In Cluster #2 (*Arbuscular Mycorrhizal* Fungi), institutions like the Spanish National Research Council (Spain) and the Zaidin Experimental Station (Spain) showcase strong cross-regional collaboration on soil–plant interactions. Similarly, Cluster #5 (Nutrient Management) highlights partnerships between King Saud University (Saudi Arabia) and Punjab Agricultural University (India), focusing on optimizing nutrient use efficiency. These thematic clusters illustrate both the diversity of research directions and the interconnected nature of global collaboration in PSM research, with institutions working together to address critical agricultural and environmental challenges.

**Table 1:** Institutions with abbreviations, full names, and corresponding countries.

Abbreviation	Institution	Country
ICAR	Indian Council of Agricultural Research	India
ICAR-IARI	ICAR-Indian Agricultural Research Institute	India
CAS	Chinese Academy of Sciences	China
UAF	University of Agriculture Faisalabad	Pakistan
CSIC	Consejo Superior de Investigaciones Científicas	Spain
CSIC-EZZ	CSIC - Estación Experimental del Zaidín	Spain
UHOH	University of Hohenheim	Germany
EKB	Egyptian Knowledge Bank	Egypt
UCA	Cadi Ayyad University of Marrakech	Morocco
UM6P	Mohammed VI Polytechnic University	Morocco
KSU	King Saud University	Saudi Arabia
UHF	Dr. Yashwant Singh Parmar University of Horticulture & Forestry	India
CSIR	Council of Scientific and Industrial Research, India	India
UT	University of Tehran	Iran
CAAS	Chinese Academy of Agricultural Sciences	China
FAFU	Fujian Agriculture and Forestry University	China

### 3.4 Author cooperation analysis

The author cooperation analysis in PSM research highlights the significant contributions and collaborative efforts of key researchers in the field (Supplementary Table S2). Hassan Etesami stands out with the highest number of publications (13) and citations (854), reflecting his central role and influence in advancing PSM studies. Adnane Bargaz (11 publications, 729 citations) and Yadav Ajar Nath (10 publications, 387 citations) are also prominent contributors, indicating active involvement in this research area. Highly cited authors such as Bernard R. Glick (1,484 citations) and Youssef Zeroual (641 citations) underscore the impactful nature of their work, despite slightly fewer publications. The data suggests a network of researchers with diverse expertise, working collaboratively to address critical challenges in the PSM domain. Emerging contributors like Olubukola Oluranti Babalola, Divjot Kour, and Hossein Ali Alikhani also highlight the growing international interest in this field. The distribution of publications and citations demonstrates a balance between well-established and newer authors, emphasizing the importance of both individual impact and collaborative synergy in driving innovation and knowledge dissemination in PSM research.

### 3.5 Keywords analysis

**Figure 5:**
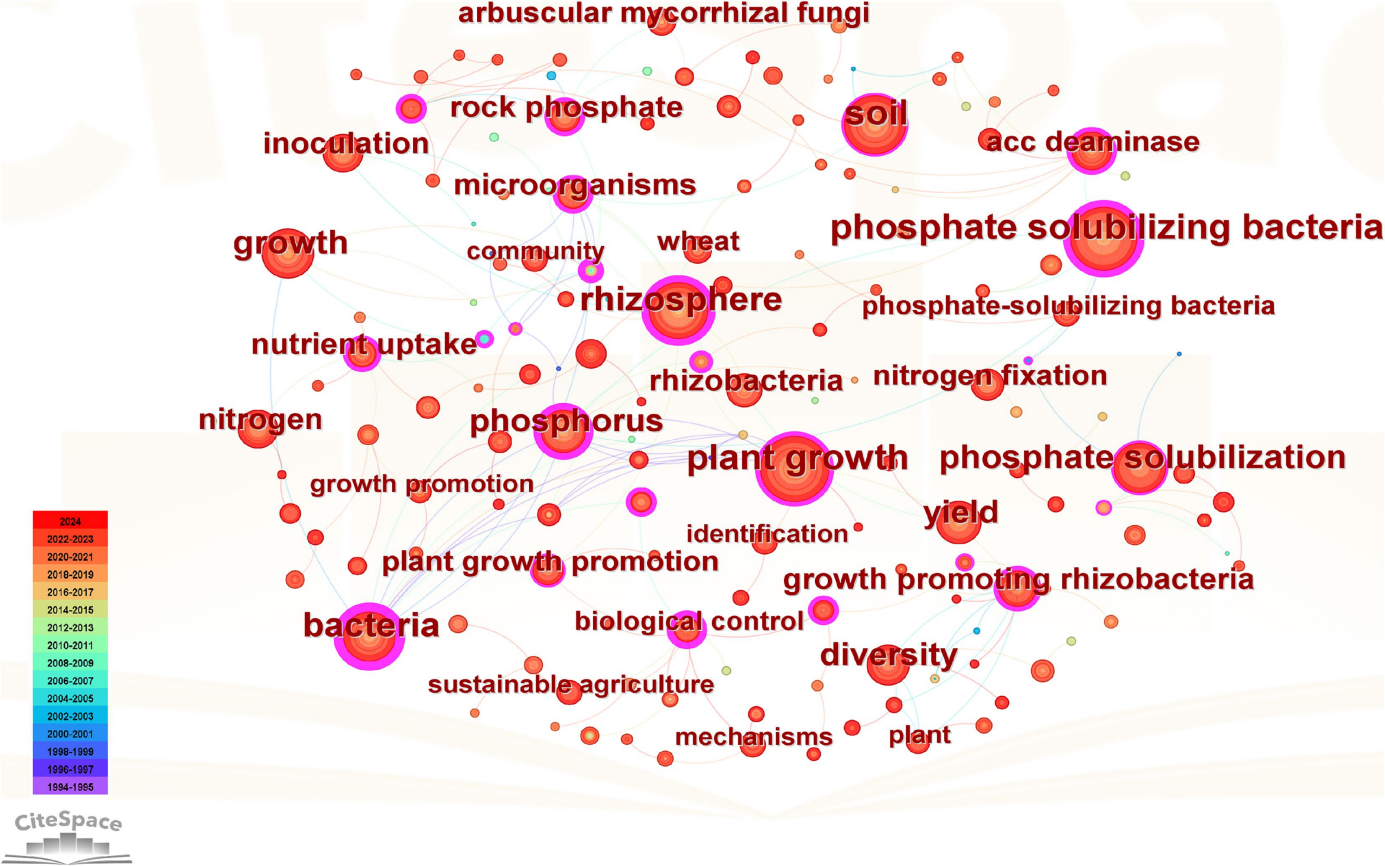
Co-occurrence network of core keywords. Each node represents a keyword, with the node size indicating its frequency of occurrence in the literature. The color of the nodes reflects the timeline of publication. Lines represent co-occurrence relationships.

Figure 5 depicts the co-occurrence network of keywords in PSM research, providing a visual representation of the field’s intellectual structure. In this network, each node corresponds to a specific keyword, with node size proportional to its frequency of occurrence in the literature (Chen, 2020). The color gradient of the nodes indicates the temporal distribution of studies, where darker red shades represent more recent research (Sabe et al., 2022). The links between nodes signify co-occurrence relationships, with thicker lines denoting stronger associations (Chen, 2020). The network highlights several core research keywords, as evidenced by the larger nodes, including “phosphate solubilizing bacteria,” “plant growth,” and “rhizosphere. “These keywords represent foundational areas of focus within PSM research. The strong connection between “phosphorus,” “rhizosphere,” and “microorganisms” suggests a sustained research emphasis on microbial-mediated phosphorus solubilization processes in the soil environment. Additionally, the association between “growth promotion” and “diversity” with mechanisms such as “nitrogen fixation” and “inoculation” reflects the integration of multifunctional microbial strategies that enhancing plant growth and nutrient acquisition. Emerging research fronts are evident in the increasing occurrence of keywords such as “nutrient uptake,” “growth promotion,” and “sustainable agriculture,” indicating a shift towards the practical application of PSM in sustainable crop production systems. The prominence of terms like “biological control” and “plant growth-promoting rhizobacteria” further underscores the expanding interest in leveraging microbial diversity for improving soil fertility and plant health.

**Figure 6:**
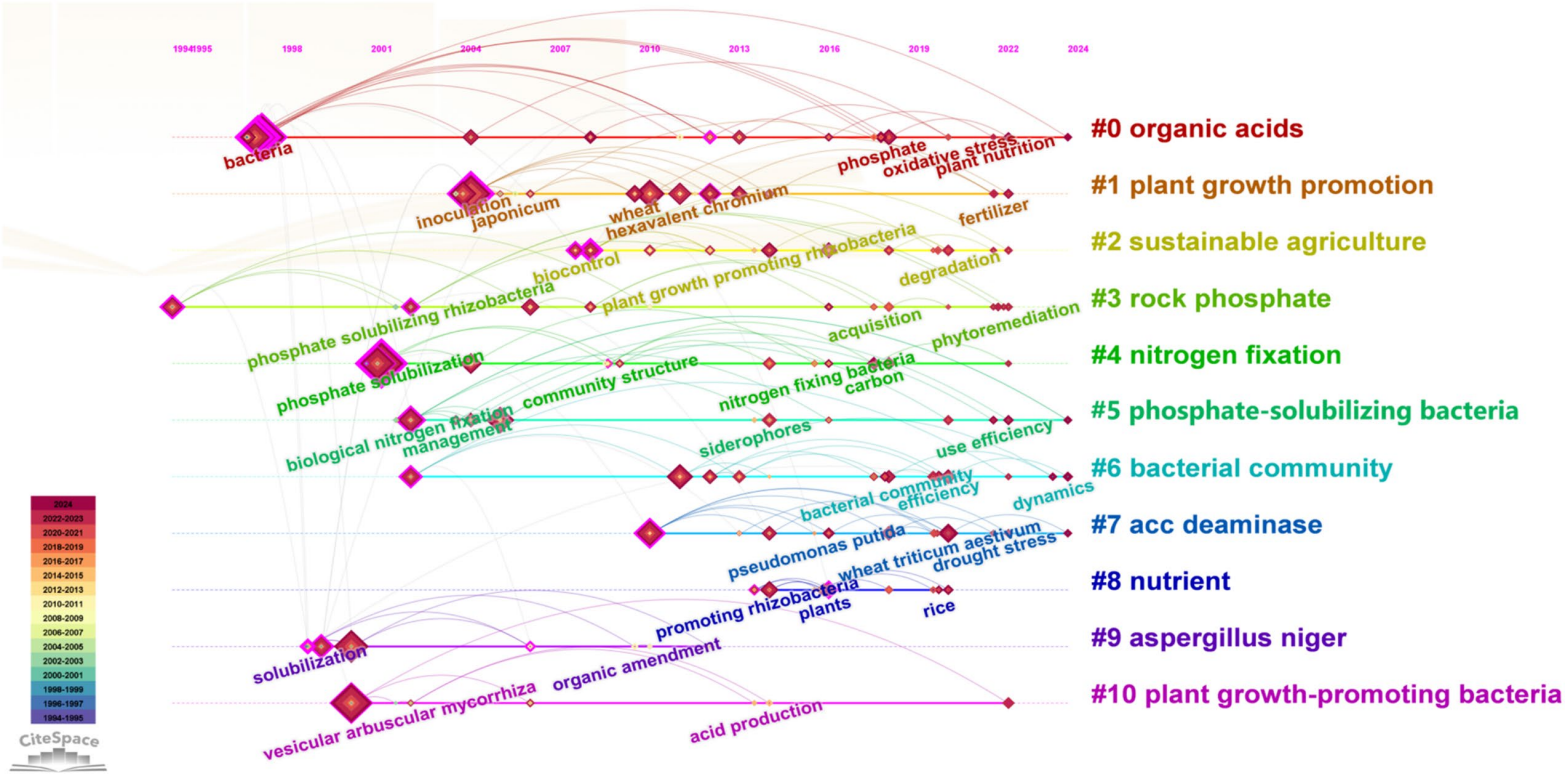
Keyword co-occurrence network timeline. The larger nodes represent high-frequency keywords in these fields, indicating the significant influence of these topics over the past few decades. The different colored lines illustrate the connections and interdisciplinary research between various themes.

Owing to the lengthy interval between the first two papers published in 1984 and 1991, a connection was made with other literature. Consequently, these two documents were not incorporated in the following analysis. Figure 6 offers a comprehensive view of the evolution of research hotspots in agricultural and environmental microbiology from 1990 to 2024, highlighting the development of key topics within the field. Early research themes, such as “organic acids,” “plant growth promotion,” and “sustainable agriculture,” emerge as prominent areas, reflecting a long-standing focus on improving agricultural productivity and sustainability. The figure also reveals the interconnected nature of research, with topics like “phosphate-solubilizing bacteria” and “nitrogen fixation” closely linked, emphasizing the crucial role of microbial processes in nutrient cycling for soil fertility. As the timeline progresses, new clusters, such as “accdeaminase” and “plant growth-promoting bacteria,” signal a shift towards mechanistic studies exploring molecular pathways involved in plant stress tolerance and growth promotion. Additionally, keywords like “phytoremediation” and “community structure” indicate a growing interest in the environmental applications of plant growth-promoting microbes, especially for soil health and pollution remediation. The color gradient, transitioning from purple (early years) to yellow (recent years), visually represents the timeline of research development, highlighting when certain topics gained prominence (Chen, 2017; Chen and Song, 2019). Clusters like “bacterial community” and “rock phosphate” further emphasize the critical role of soil microbes and microbial diversity in enhancing soil health and phosphorus availability, which are vital for sustainable agricultural practices. Overall, this visualization provides a detailed overview of how research within soil microbiology and plant growth has evolved, focusing on microbial processes, soil health, and environmental concerns, alongside mechanistic studies aimed at addressing global agricultural challenges like climate change and soil degradation.

**Figure 7:**
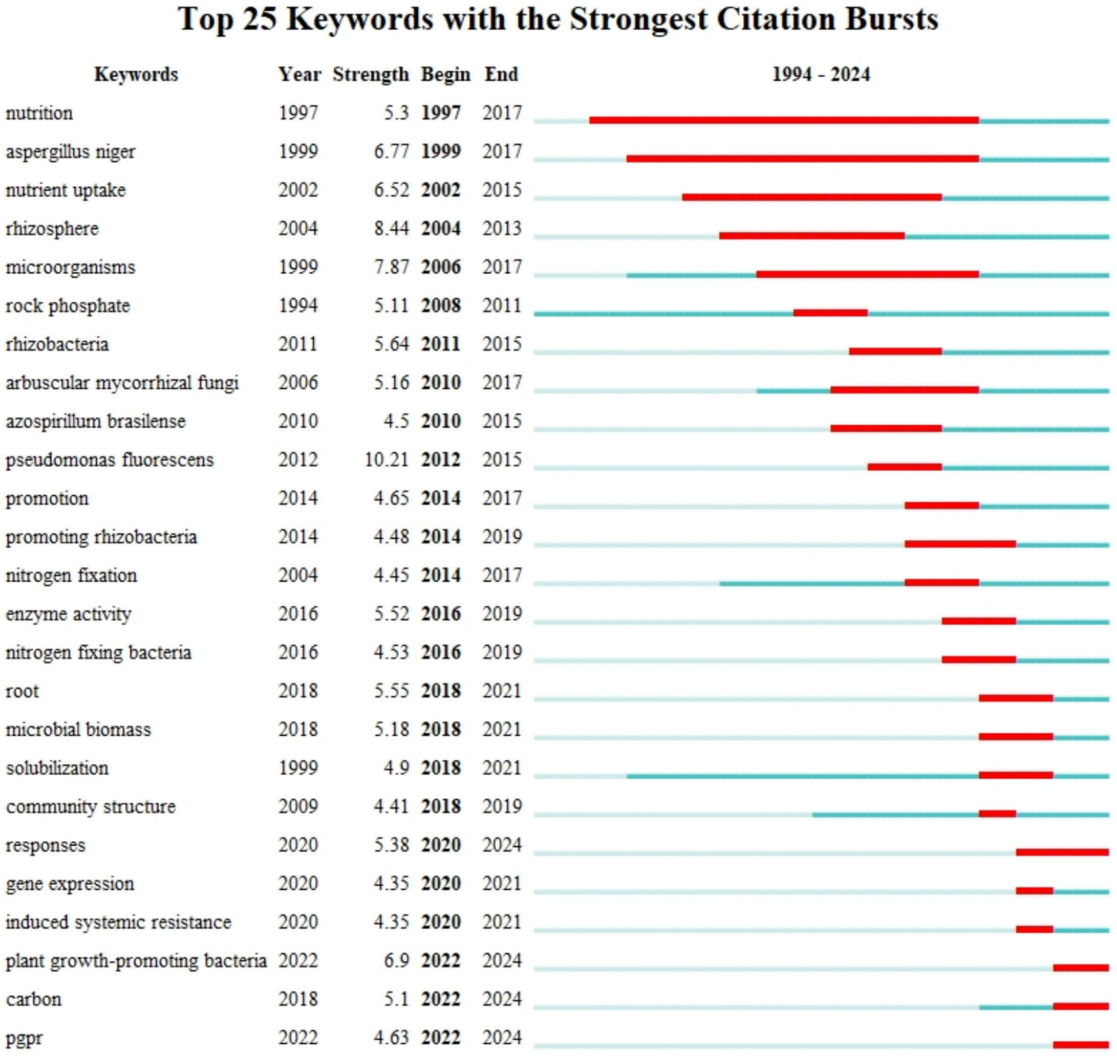
Top 25 keywords with the strongest citation bursts from 1994 to 2024.

Figure 7 illustrates the 25 keywords with the strongest Citation Bursts in the academic literature. Adjacent to each keyword is the year of its citation surge, intensity, start and end times, and the corresponding time period (Hou et al., 2018). Each keyword is accompanied by its surge year, intensity, duration, and corresponding time period. Early bursts like “nutrition” (1997) and “*Aspergillus niger*” (1999) reflect sustained interest in microbial roles in plant nutrient uptake, using traditional microbiological methods. Keywords from the early 2000s, such as “nutrient uptake” and “rhizosphere,” indicate growing research on plant-microbe interactions, transitioning towards molecular techniques. Later bursts like “*arbuscular mycorrhizal* fungi” (2006) and “*azospirillum brasilense*” (2010) highlight increased focus on beneficial fungi and nitrogen-fixing bacteria, enhanced by metagenomics and sequencing technologies. Recent bursts, including “promoting rhizobacteria” (2014) and “nitrogen fixation” (2016), signal a shift towards microbial solutions for soil fertility and crop yields, utilizing omics technologies. Keywords like “microbial biomass” (2018) and “solubilization” (2019) emphasize microbial roles in phosphate solubilization, supported by advanced analytical methods. The most recent bursts, such as “gene expression” and “induced systemic resistance” (2020–2024), suggest a focus on molecular mechanisms of plant-microbe interactions, often using gene-editing technologies like CRISPR. Overall, the data highlights a shift in PSM research from basic functions to applied solutions, with increasing attention on enhancing soil health and sustainable farming through microbial interventions. This evolution from conventional to advanced techniques offers insights into modern approaches suitable for current and future studies.

### 3.6 Reference and journal co-citation analysis

The top 10 co-citation articles in PSM research filed are listed in Table 2. The most influential article, “Phosphate-solubilizing bacteria and their role in plant growth promotion” by Rodríguez and Fraga (1999), with 3,321 co-citation, underscores its foundational role in linking PSM to plant growth enhancement. Sharma et al. (2013) work on sustainable phosphorus management, ranks second with 2,816 citations, highlighting the pivotal role of PSM in addressing phosphorus deficiency in agricultural soils. Other significant studies, such as Nautiyal (1999) and Pikovskaya (1948), focus on an efficient microbiological growth medium and microbial contributions to soil nutrient mobilization, achieving 2,291 and 2,158 citations, respectively. These articles collectively demonstrate high total link strengths, with values exceeding 1900, indicating their substantial connectivity and influence within the research community. Top 20 journals by number of publications from 1,622 articles are listed in Supplementary Table S3. A total of 1,622 articles were published across 472 journals, with 40 journals publishing more than 10 articles, as illustrated in Supplementary Figure S3.

**Table 2:** Top ten co-citation articles in PSM research.

Rank	Article title	Authors	Co-citation	Published year	Reference
1	Phosphorous solubilizing bacteria and their role in plant growth promotion	Rodríguez et al.	3321	1999	Rodríguez and Fraga (1999)
2	Phosphate solubilizing microbes: sustainable approach for managing phosphorus deficiency in agricultural soils	Sharma et al.	2816	2013	Sharma et al. (2013)
3	An efficient microbiological growth medium for screening phosphate solubilizing microorganisms	Nautiyal	2291	1999	Nautiyal (1999)
4	Phosphorous solubilizing bacteria from subtropical soil and their tricalcium phosphate solubilizing abilities	Chen et al.	2158	2006	Chen et al. (2006)
5	Mobilization of phosphorous in soil in connection with the vital activity of some microbial species	Pikovskaya	2123	1948	Pikovskaya (1948)
6	Microbial phosphorus solubilization and its potential for use in sustainable agriculture	Alori et al.	2007	2017	Alori et al. (2017)
7	Universal chemical assay for the detection and determination of siderophores	Schwyn and Neilands	1788	1987	Schwyn and Neilands (1987)
8	Plant growth-promoting bacteria: mechanisms and applications	Glick	1626	2012	Glick (2012)
9	Colorimetric estimation of indoleacetic acid	Gordon and Weber	1103	1951	Gordon and Weber (1951)
10	A modified single solution method for the determination of phosphate in natural waters	Murphy and Riley	1037	1962	Murphy and Riley (1962)

### 3.7 Subject categories co-occurrence analysis

**Figure 8:**
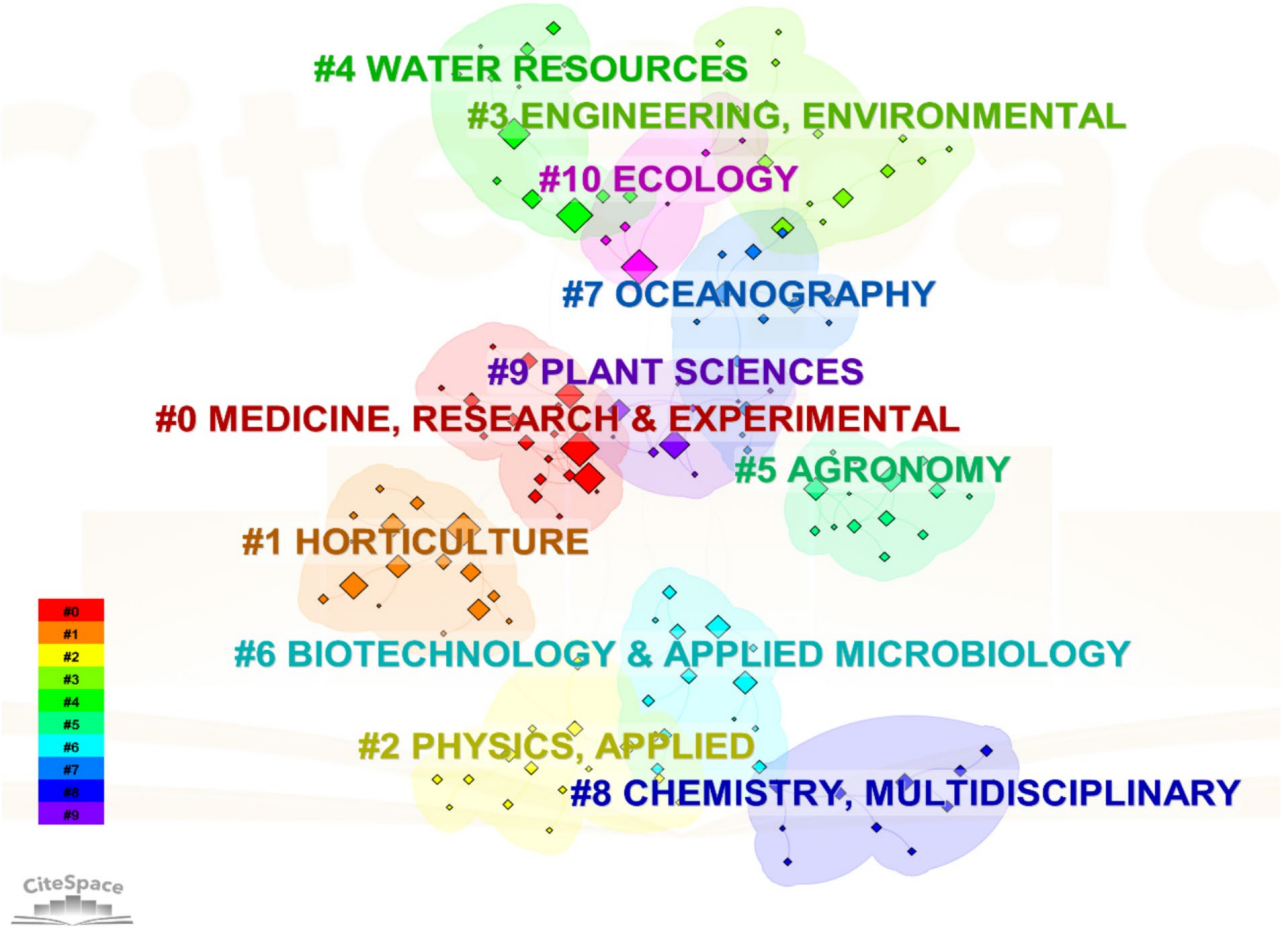
Subject co-occurrence analysis in the scientific research field of PSM. Different colors represent distinct clusters research themes. Each color typically indicates a specific area of study.

The co-occurrence analysis of subject categories contributes to the advancement of research frontiers and the identification of interdisciplinary characteristics within specific knowledge fields (Li et al., 2019). Utilizing cluster analysis in CiteSpace software, we identified that research on PSM is both multidisciplinary and interdisciplinary (Figure 8), generating seven distinct clusters. This research encompasses various fields, including medicine, research and experimental, horticulture, physics, engineering environment, water resources, agronomy, biotechnology and applied microbiology, oceanography, chemistry, plant sciences, and ecology.

## 4 Discussion

Research on PSM from 1984 to 2024 illustrates a remarkable evolution in focus, with growing contributions from diverse institutions and countries, reflecting regional priorities and global challenges. Early studies primarily focused on the fundamental mechanisms of phosphate solubilization. Rodríguez and Fraga (1999) emphasized that the secretion of organic acids, such as citric and oxalic acids, is a key mechanism, as these acids lower soil pH and promote phosphate solubilization. Illmer and Schinner (1992) demonstrated that environmental conditions (e.g., pH, temperature, and carbon sources) significantly influence the efficiency of bacterial phosphate solubilization. Kim et al. (1997) identified acid phosphatase and phospholipase as crucial enzymes for the mineralization of organic phosphorus, converting it into plant-available forms. Further molecular studies revealed that certain bacteria (e.g., *Pseudomonas*) possess genes regulating the synthesis of organic acids and extracellular enzyme secretion, which are controlled by environmental phosphate levels (Goldstein, 1986). Additionally, microbial interactions, such as the synergistic relationship between phosphate-solubilizing bacteria and AMF, can enhance plant phosphorus uptake (Zheng et al., 2024; García-Berumen et al., 2025). These studies, conducted primarily in Europe and North America, laid a theoretical foundation for developing PSM-based microbial fertilizers. Despite the limited number of publications and modest growth in the field, studies explored the mode of action of PSM (Toro et al., 1997), their diversity (De Souza et al., 2000), and their interactions with plant roots (Sharma, 2003).

In the following decades, PSM research expanded globally, with notable contributions from countries like India, China, and Brazil. Key institutions, including the Indian Council of Agricultural Research and the Chinese Academy of Sciences, advanced PSM applications in agriculture and soil management. Studies emphasized PSM synergies with other microorganisms. Kohler et al. (2007) and Egamberdiyeva and Höflich (2003) highlighted interactions with AMF and plant growth-promoting rhizobacteria, demonstrating enhanced nutrient uptake and plant growth, particularly in tropical and subtropical regions. During this period, policy support in many countries began to emerge, recognizing the role of biofertilizers in sustainable agriculture. For instance, India’s National Mission for Sustainable Agriculture and China’s Green Development Policy encouraged the use of biofertilizers like PSM to improve soil fertility and reduce chemical fertilizer dependency (Jianbo Shen et al., 2024; Kaur et al., 2024).

Thematic priorities diversified to address nutrient management, ecosystem restoration, and microbial interactions. Research focused on PSM roles in improving soil health, plant growth, and productivity under environmental stress (Rodríguez and Fraga, 1999; Khan et al., 2007). Some studies demonstrated field applications for phosphorus-deficient soils, further supporting the potential of PSM in improving soil fertility and aiding in the bioremediation of heavy metal contamination, particularly lead, cadmium, and chromium (Sharma et al., 2013; Guo et al., 2021a; Guo et al., 2021b; Li et al., 2022). Recent studies have expanded this understanding by examining PSM performance across diverse ecosystems, climates, and soil types. For instance, PSM applications in arid and semi-arid regions have shown promise in mitigating drought stress and enhancing soil fertility (Rodríguez and Fraga, 1999). In saline soils, certain halotolerant PSM strains have effectively solubilized phosphorus, contributing to plant resilience and productivity (Kushwaha et al., 2024). Similarly, PSM adapted to acidic soils have improved phosphorus availability and crop yields in tropical regions (Khan et al., 2007). Synergistic interactions with other microbes, such as nitrogen-fixing bacteria (Zaidi et al., 2009) and potassium-solubilizing microorganisms (Yadav, 2022), improved soil nutrient bioavailability and supported ecological restoration on a larger scale. These research advancements aligned with global policy initiatives, such as the European Union’s Farm to Fork Strategy, which promotes sustainable agricultural practices by supporting microbial fertilizers through funding and regulatory frameworks (Kour et al., 2020; Dasgupta et al., 2021).

The post-2020 period has seen a global shift in PSM research, driven by pressing challenges such as climate change, environmental stresses, and the need for ecological resilience. This trend is marked by growing contributions from countries like India, China, and the United States, with key institutions such as the Indian Institute of Soil Science, the Chinese Academy of Agricultural Sciences, and the United States Department of Agriculture advancing this field. Some research on PSM has concentrated on their utilization in alleviating environmental stresses induced by climate change, particularly under drought and salt conditions (Kumari et al., 2023); the cooperative interactions between PSM and other microorganisms, enhancing nutrient acquisition and pathogen resistance in crops (Phour and Sindhu, 2023); genomic and metabolomic investigations to elucidate phosphate solubilization mechanisms and identify highly efficient strains (Kumari et al., 2023). In addition, through collaborative efforts between academia and industry, progress has been made in industrial applications that promote psm in sustainable agricultural practices (Phour and Sindhu, 2023). This has a positive impact on soil health and the carbon cycle and can contribute to the sustainable development of agro-ecosystems. Moreover, public-private partnerships have facilitated the commercialization of PSM-based products, bridging scientific research and practical agricultural applications (Verma et al., 2019). However, challenges such as production standardization, quality control, and regulatory disparities across regions must be addressed to maximize PSM’s potential in sustainable agriculture (Gopalakrishnan et al., 2015).

Significant progress has been made in the study of PSM from 1984 to 2024, but several challenges remain, particularly concerning their integration into sustainable ecosystem management. Key issues include: (1) limited understanding of molecular and enzymatic mechanisms underlying phosphate solubilization (Rodríguez and Fraga, 1999; Raghothama and Karthikeyan, 2005). (2) The environmental adaptability of PSM remains a critical yet insufficiently explored area, particularly given their variable performance across diverse ecosystems, climatic conditions, and soil types. The underlying mechanisms by which temperature fluctuations, soil pH, moisture levels, and nutrient availability affect PSM survival, colonization, and phosphate-solubilizing efficiency have not been thoroughly elucidated (Alori et al., 2017). (3) underdeveloped multifunctional traits, such as nitrogen fixation and plant growth promotion (Adesemoye et al., 2009). (4) Insufficient research on PSM interactions with soil microbes, particularly in nutrient cycling and ecosystem stability (Richardson and Simpson, 2011; Bashan et al., 2013). (5) Research on the long-term stability and ecological safety of PSM is limited, particularly regarding their impact on native microbial communities and soil functions (Vassilev et al., 2013). (6) Inconsistent policies and regulations across regions hinder the large-scale commercialization and adoption of PSM-based biofertilizers. The absence of unified standards for production quality, application methods, and environmental safety creates barriers to industry adoption and farmer use (Yadav and Yadav, 2024). Addressing these challenges will be vital for advancing PSM-based solutions for sustainable agriculture and ecosystem restoration.

## 5 Conclusion

This bibliometric study analyzed 1,662 publications on PSM spanning 1984–2024, revealing distinct evolutionary phases in research focus and applications. The initial phase (1984–2008) concentrated on isolating and characterizing PSM, emphasizing their biochemical mechanisms for phosphorus solubilization. The subsequent period (2009–2017), the research expanded to agricultural applications, exploring PSM as biofertilizers within organic farming systems. The recent phase (2018 to present) reflects an increased focus on ecological restoration, soil pollution control, and their role in addressing global challenges such as climate change and nutrient resource recycling.

Despite significant progress, challenges in PSM remain, such as limited understanding of their mechanisms across environments, poor integration of genomic and metabolic analyses, and inconsistent field outcomes due to environmental factors. Future research should focus on: **(1) Unraveling Functional Mechanisms**: Using omics approaches to uncover metabolic pathways and regulatory networks involved in phosphate solubilization. **(2) Environmental Adaptability**: Investigating PSM resilience and adaptability under varying environmental stresses, such as drought, salinity, and extreme temperatures, to ensure consistent field performance. **(3) Multifunctional Microbial Design**: Engineering PSM with additional functionalities such as nitrogen fixation and stress tolerance using advanced biotechnological tools. **(4) Microbial Interactions**: Exploring how PSM influence and are influenced by native soil microbiomes to maintain ecological balance. **(5) Biofertilizer Development**: Focusing on scalable production technologies, formulation stability, and delivery methods to improve the commercial viability of PSM-based biofertilizers. **(6) Policy and Research Integration:** Promoting interdisciplinary research and policy integration to support the sustainable application of PSMs in agriculture and ecological restoration. By expanding research in these directions, PSM can be more effectively harnessed to address global challenges such as soil degradation, food insecurity, and climate change. This comprehensive approach will support resilient agricultural systems and accelerate progress toward global sustainability targets.

## Data Availability

The datasets presented in this study can be found in online repositories. The names of the repository/repositories and accession number(s) can be found at: https://data.mendeley.com/drafts/xrp4hp58gs.
