# Supplementary material for: Phosphate-solubilizing microorganisms for soil health and ecosystem sustainability: a forty-year scientometric analysis (1984–2024)
Source: Front Microbiol. 2025 Feb 19;16:1546852. doi: 10.3389/fmicb.2025.1546852 (PMC11879999; doi:10.3389/fmicb.2025.1546852)
Supplement: Supplementary file 1 [file Data_Sheet_1.docx]

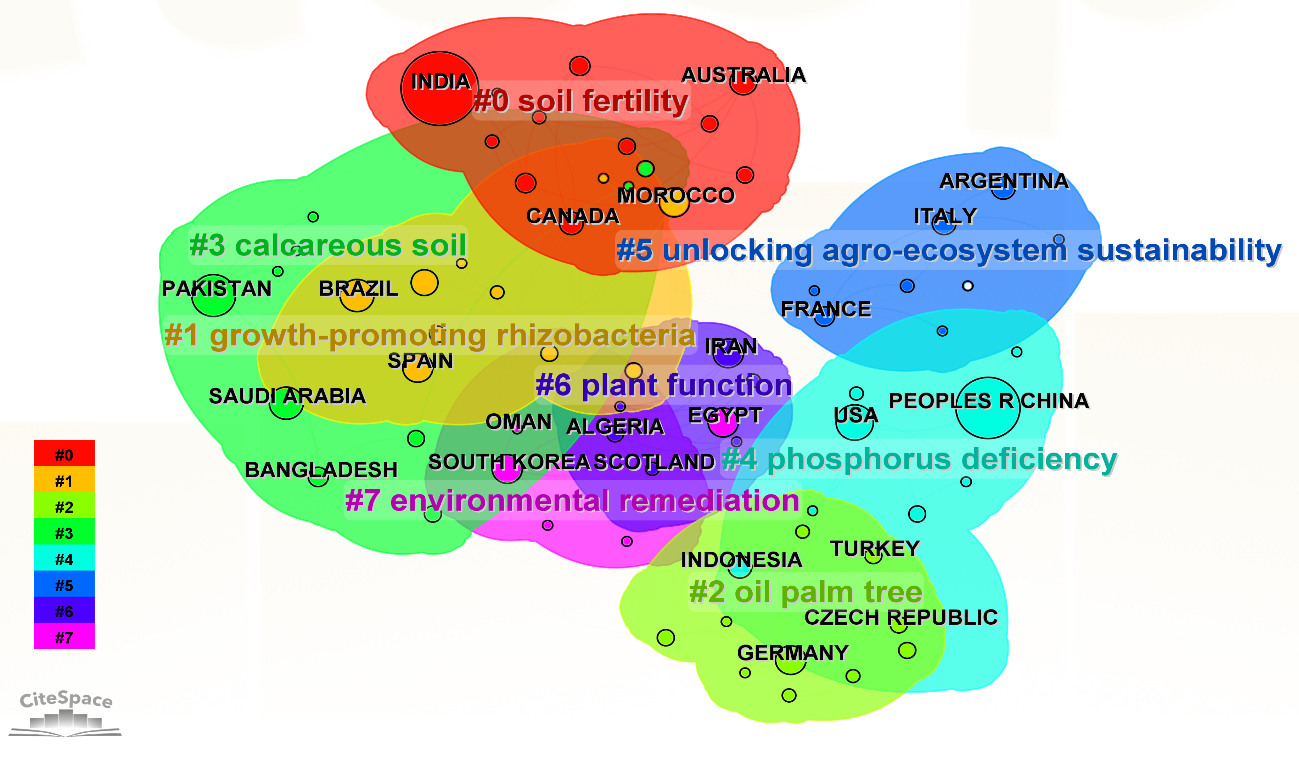


**Fig. S1 The network of contributions by country and research topic**

Colors represent distinct research clusters, each corresponding to a specific thematic focus in the field of PSM research. Nodes indicate countries or regions actively contributing to PSM research, with larger nodes reflecting higher levels of publication output or influence. Lines between nodes represent collaborative relationships, where thicker lines signify stronger or more frequent cooperation between regions or institutions.


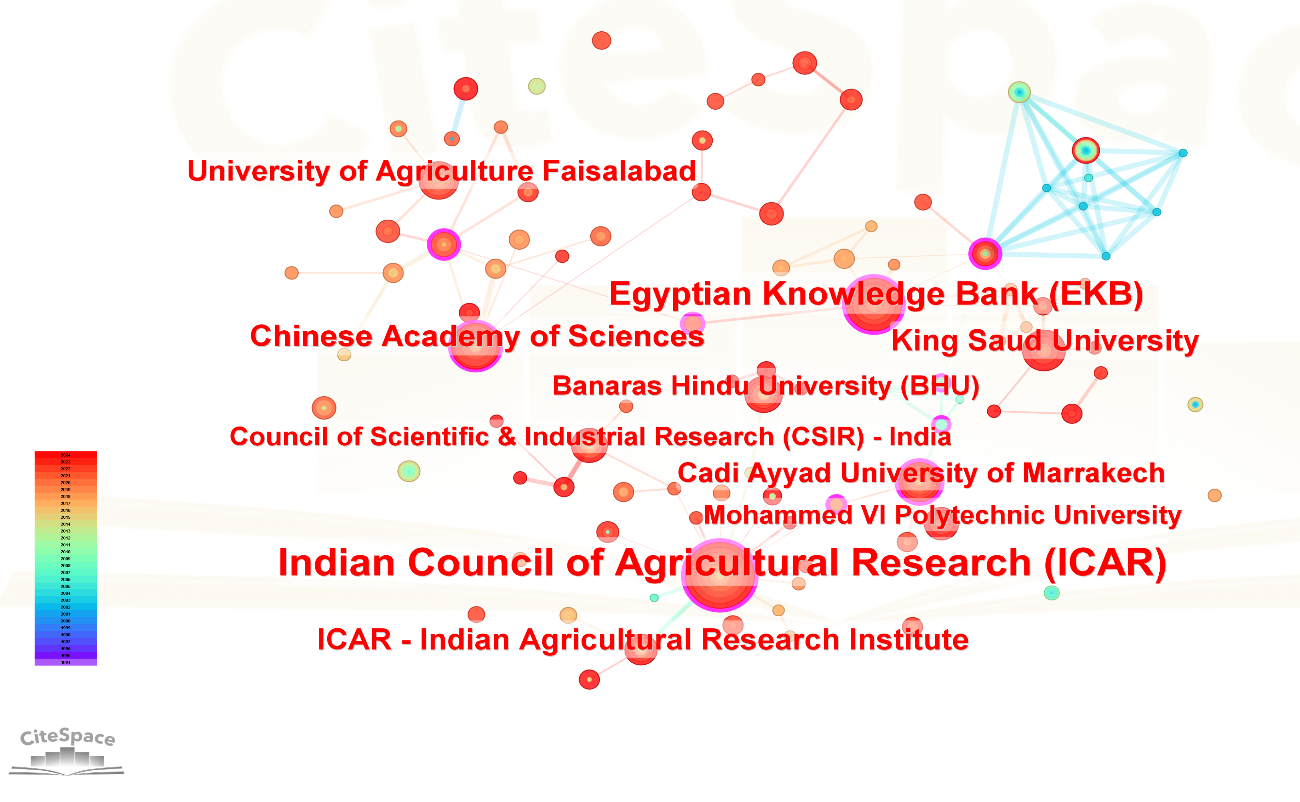


**Fig. S2 Network analysis of research institutions in PSM field**

Nodes represent different countries involved in the collaboration network, with their size indicating the level of participation in international cooperation. Color indicates the strength or frequency of collaborations, with larger nodes suggesting more frequent partnerships. Lines between the nodes represent collaborative relationships between countries, and the darker the lines indicate more frequent collaborations.


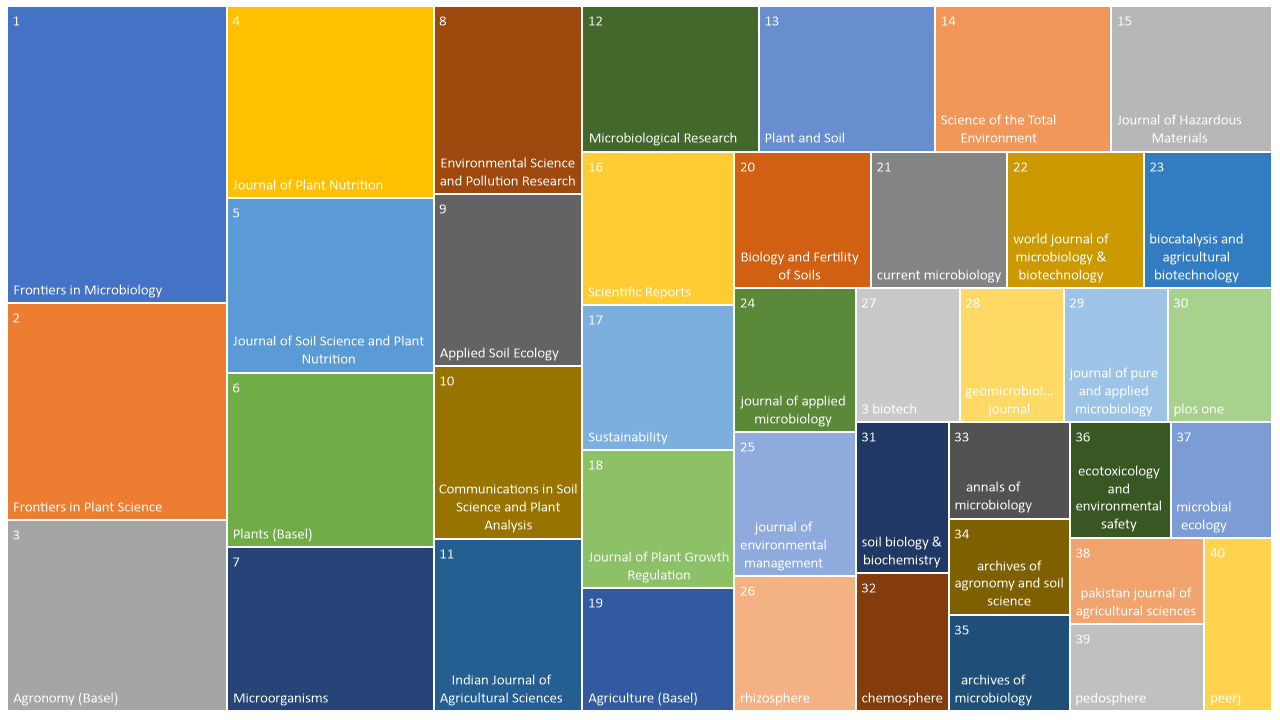


**Fig. S3 Tree graph of the top 40 journals by number of published articles**

Colors represent different journals. Block sizes indicate the relative contribution or number of publications from each journal, with larger blocks representing higher publication counts.

**Table S1 Top 20 countries in the number of publications**

| Rank | Country | Count | Centrality | Year |
| --- | --- | --- | --- | --- |
| 1 | INDIA | 477 | 0.00 | 1999 |
| 2 | CHINA | 343 | 0.34 | 2005 |
| 3 | PAKISTAN | 130 | 0.85 | 2004 |
| 4 | USA | 91 | 0.11 | 2004 |
| 5 | BRAZIL | 78 | 0.08 | 2002 |
| 6 | SAUDI ARABIA | 67 | 0.39 | 2004 |
| 7 | SPAIN | 65 | 0.08 | 1997 |
| 8 | IRAN | 61 | 0.00 | 2010 |
| 9 | MOROCCO | 57 | 0.45 | 2008 |
| 10 | SOUTH KOREA | 56 | 0.08 | 2003 |
| 11 | EGYPT | 55 | 0.19 | 2004 |
| 12 | GERMANY | 54 | 0.32 | 2002 |
| 13 | AUSTRALIA | 48 | 0.19 | 1999 |
| 14 | MEXICO | 43 | 0.00 | 2001 |
| 15 | INDONESIA | 32 | 0.00 | 2000 |
| 16 | CANADA | 31 | 0.11 | 2002 |
| 17 | ITALY | 30 | 0.08 | 2003 |
| 18 | FRANCE | 26 | 1.07 | 2003 |
| 19 | ARGENTINA | 25 | 0.00 | 2007 |
| 20 | BANGLADESH | 24 | 0.04 | 2016 |

**Table S2: The Top 20 Authors with the Most Publications in the PSM Field**

| Rank | Author | Count | Year | documents | citations |
| --- | --- | --- | --- | --- | --- |
| 1 | Etesami, Hassan | 13 | 2017 | 13 | 854 |
| 2 | Bargaz, Adnane | 11 | 2018 | 11 | 729 |
| 3 | Yadav, Ajar Nath | 10 | 2020 | 10 | 387 |
| 4 | Zeroual, Youssef | 10 | 2018 | 10 | 641 |
| 5 | Babalola, Olubukola Oluranti | 9 | 2018 | 9 | 288 |
| 6 | Kour, Divjot | 9 | 2020 | 9 | 382 |
| 7 | Alikhani, Hossein Ali | 8 | 2018 | 8 | 263 |
| 8 | Glick, Bernard R | 8 | 2017 | 8 | 1484 |
| 9 | Hafidi, Mohamed | 8 | 2008 | 8 | 352 |
| 10 | Khan, Mohammad Saghir | 8 | 2007 | 8 | 1026 |
| 11 | Kumar, Anil | 8 | 2009 | 8 | 44 |
| 12 | Li, Zhen | 8 | 2016 | 8 | 253 |
| 13 | Naveed, Muhammad | 8 | 2016 | 8 | 223 |
| 14 | Ahmad, Maqshoof | 7 | 2016 | 7 | 202 |
| 15 | Datta, Rahul | 7 | 2020 | 7 | 342 |
| 16 | Fahad, Shah | 7 | 2019 | 7 | 448 |
| 17 | Kaur, Tanvir | 7 | 2021 | 7 | 206 |
| 18 | Lyamlouli, Karim | 7 | 2018 | 7 | 540 |
| 19 | Ouhdouch, Yedir | 7 | 2008 | 7 | 350 |
| 20 | Shahid, Muhammad | 7 | 2016 | 7 | 169 |

**Table S3:** **Top 20 Journals by Number of Publications from 1622 Articles**

| Rank | Source | Documents | Citations | Total link strength |
| --- | --- | --- | --- | --- |
| 1 | Frontiers in Microbiology | 56 | 2494 | 508 |
| 2 | Frontiers in Plant Science | 41 | 1564 | 356 |
| 3 | Agronomy (Basel) | 36 | 928 | 213 |
| 4 | Journal of Plant Nutrition | 34 | 364 | 140 |
| 5 | Journal of Soil Science and Plant Nutrition | 31 | 1080 | 274 |
| 6 | Plants (Basel) | 31 | 622 | 183 |
| 7 | Microorganisms | 29 | 548 | 253 |
| 8 | Environmental Science and Pollution Research | 24 | 328 | 104 |
| 9 | Applied Soil Ecology | 22 | 965 | 149 |
| 10 | Communications in Soil Science and Plant Analysis | 22 | 269 | 108 |
| 11 | Indian Journal of Agricultural Sciences | 22 | 116 | 19 |
| 12 | Microbiological Research | 22 | 1273 | 204 |
| 13 | Plant and Soil | 22 | 2222 | 247 |
| 14 | Science of the Total Environment | 22 | 803 | 128 |
| 15 | Journal of Hazardous Materials | 20 | 743 | 77 |
| 16 | Scientific Reports | 20 | 547 | 122 |
| 17 | Sustainability | 19 | 181 | 73 |
| 18 | Journal of Plant Growth Regulation | 18 | 265 | 113 |
| 19 | Agriculture (Basel) | 16 | 389 | 117 |
| 20 | Biology and Fertility of Soils | 16 | 2046 | 164 |
